# Some Cases of Diabetes

**Published:** 1910-11-19

**Authors:** Nestor Tirard


### HOSPITAL CLINICS.

### SOME CASES OF DIABETES.

By NESTOR TIRARD, M.D., F.R.C.P.

A Lecture delivered at the Polyclinic, Chenies Street, W.C., on Monday, October 10, 1910.*

WITH regard to these complications, the first is albuminuria. The case to which I referred is a man aged fifty-four, who was admitted in an unconscious condition. It is interesting, in view of what follows, that the father died at the age of seventy of diabetes. According to the account which the friends gave, the patient had had splendid health until March last year. Then he had a smart attack of gastric influenza, whatever that may have been, and had never been well since. He had had much worry since a brother's death, and had had a great deal of overwork lately; he was a printer on night work. On May 26 of this year he came home from work with severe headache. On the 27th the headache continued, and he kept to his bed, and there was delirium in the evening. On the 28th he was unconscious at intervals, on the 30th he was worse, and on the 31st completely unconscious. He was admitted on June 1, with unconsciousness, incontinence of urine, and incontinence of fæces; with a subnormal temperature and a pulse of 92.

### THE DIAGNOSIS.

Our difficulty, of course, turned on what was the cause of the unconsciousness. One pupil was a little dilated at the beginning, but the pulse tension was high, the cardiac impulse was outside the normal situation, and the heart sounds had the exaggerated systolic sound over the cardiac impulse, and an exaggerated, reduplicated second sound over the base of the heart, which we are accustomed to associate with chronic albuminuria. So far, we had no urine to guide us; it was some days before we obtained a specimen, and the patient was treated at once as a case of uræmia. When we obtained a small specimen of urine it was found that it reduced Fehling's solution, that it gave ordinary tests for sugar, and that it contained albumen. A little later we found that the knee jerks were absent. On June 5 he was delirious, noisy, troublesome. On June 16 he regained consciousness little by little. The knee jerks remained absent to the end, and that indication belongs rather to diabetes than to uræmia. When we obtained further specimens of the urine, the specific gravity was found to vary from 1020 to 1027, and it gave the reaction for both albumen and sugar.

### TREATMENT.

We modified our treatment when we found sugar; instead of treating him with croton oil and purgatives, vapour baths, etc., we proceeded to attempt to render the urine alkaline. We gave salicylate of soda and bicarbonate of soda. The patient went out towards the end of July with a clear memory, and able to walk quite well, but still with a trace of sugar and a little albumen. I do not quite know in that case whether the unconsciousness was primarily uræmic or primarily diabetic. That leads me to say that in diabetes you must not always expect to find a very high specific gravity. Urinometers are, as you know, marked on one side zero to 30, 40, onwards; on the other side there are mystic marks and the word "diabetes"; as though if the diabetic mark on the urinometer were not reached, the case was all right. I have long found that students do not recognise the fact that in gouty people with glycosuria, and even in gouty people with true diabetes, the specific gravity may be comparatively low, that you may find what is to all intents and purposes a relatively large amount of sugar by chemical tests with a low specific gravity, owing to the reduction of the other solids, just as you find a low specific gravity in ordinary cases of gout with contracted kidney, and an increased quantity of water.

### ALKALINE URINE.

Another feature which I want to dwell upon arises out of the theoretical considerations of this case. You will find, I think, in all theoretical discussions connected with the treatment of diabetes or its management, that with the onset of nervous symptoms, of coma, and even severe headache or with the presence of acetone or diacetic acid in the urine, if the urine gives a red colour with perchloride of iron you are advised to make the urine alkaline as quickly as you can. It is quite good advice, but not quite so easy to carry out. Those who have not watched closely would be surprised at the difficulty often experienced in reducing the acidity, and in obtaining an alkaline reaction. I have the record of a patient who was kept under observation over a month, taking large doses of alkalies the whole time, and yet there was no alkalinity of the urine. Once it was neutral, and on all other occasions it remained acid.

### DRUGS IN TREATMENT.

Now, with regard to treatment, I am in the habit of employing large doses of alkali. I consider it important to diminish the acidity, as that acidity is usually due to one of the fatty acids. The patient is put on 30 grains of bicarbonate of soda three times a day. If there are indications of acetone, or diacetic acid in the urine, or if there is any tendency to headache, the alkali administered is largely increased, and I have been in the habit of giving simultaneously fairly large doses of sodium salicylate. It was under this treatment that the first patient I referred to lost her sugar and gained weight. When there is headache, drowsiness, or apathy it is advisable to increase the dose. For the second case I mention a modification of this treatment was adopted. Some of the French writers have employed massive doses of salines in the treatment of diabetes. I have always employed average doses of

* The first part of this lecture was reported in THE HOSPITAL of November 12, p. 191.

magnesium sulphate. In this patient, who was very bad with headache and drowsiness and had lost much weight, I employed massive doses of magnesium sulphate. I gave 35 oz. of Hunyadi Janos. Had it not been tried by others I confess I should have been alarmed as to the result. But he only passed two liquid motions, and there was an immediate fall in the amount of sugar, with immediate relief of the nervous system. When the patient left the hospital the sugar was still smaller in amount than before. Under similar conditions I propose to try it again, although I did not obtain the same benefits as Dr. Philip, for he found the sugar disappeared entirely. When giving that amount of Hunyadi Janos, the patient was only allowed, for the twenty-four hours, a pint of milk and as much water as he cared to take.

Time compels me to sum up. The modifications of diet I recommend are the removal of sugar from and the diminution of carbohydrates in the food. At the same time the symptoms must be closely watched; any drowsiness, any indication of great loss of weight must be a hint to return to some carbohydrate. In the case of a patient whose urine was free from sugar when she left the hospital, she had for days been taking 2 to 4 oz. of ordinary white bread, and the sugar remained absent notwithstanding. When there had been a slight increase in the sugar we reduced the bread to 1 oz. In spite of what has been written on the subject, I am not generally in favour of administering either opium, morphine, or codeine. Many observers maintain that after you have reached the lowest daily amount of sugar, with changes of diet, you may make sugar disappear from the urine by giving opium or codeine. In my experience I have found great difficulty in being certain that the drowsiness which often ensued was not due to the opium rather than to the disease. I hold that the average patient is better without morphine than with it; it is better not to encourage him to lean on morphine.

Air-hunger.

With regard to the treatment of air-hunger, or of severe coma, I have seen air-hunger develop in a patient whose urine while in the hospital had neither a large amount of sugar nor a very high specific gravity. She had rapid loss of weight, and was an uncomfortable patient, who refused to follow any form of dietary, and finally had to be sent out. To my sorrow, I was afterwards asked to see her with her medical man with an acute onset of air-hunger as the result of acidosis. Another case of air-hunger was one where this symptom was the first indication of diabetes. I think it is unique in my experience. The person believed himself to be perfectly well, and was going on with active work until suddenly seized with dyspnœa of acute character. He then came under medical observation. The freedom of the entry of air and the absence of physical signs led at once to an examination of the urine, and sugar was found. As with coma, you have to treat the case as one of immediate danger; I doubt whether anything is going to do any good. Usually, the end comes in twenty-four to forty-eight hours from the onset of the symptoms. I feel sure that the only form of treatment likely to be successful will be one which removes the toxin from the blood. It is no use merely injecting saline solution, nor merely giving alkalies in large quantities by the mouth, or even subcutaneously or intravenously, unless at the same time some of the toxin is removed by venesection.

I find myself at the end of my time with many more points still to put before you, but not until I began to prepare my material did I realise that there was so much ground to cover.

